# Supplementary material for: Content-rich biological network constructed by mining PubMed abstracts
Source: BMC Bioinformatics. 2004 Oct 8;5:147. doi: 10.1186/1471-2105-5-147 (PMC528731; doi:10.1186/1471-2105-5-147)
Supplement: Additional File 2 — The original results of the above study (non-essential files are deleted to keep the file size under the limit set by BMC bioinformatics). [file 1471-2105-5-147-S2.bz2 › chilibotAdditionalFile2/dip20/208ID8846021E708/html/ITGA1_ITGB1.html]

 


 **ITGA1** and **ITGB1** 
  
Found 245 abstracts in PubMed, retrieved 20.  
 

 What does Google say? 
 PDF only 
| .edu only 

---

**Interactive relationship** (e.g. stimulation, inhibition, etc)

**Non-interactive relationship** (e.g. studied together, co-existance, homology, etc.)

- ATG also contained functional antibodies to the beta1 integrin CD49d CD29  [ **ITGB1** ]  VLA  [ **ITGA1** ]  4, the alpha4beta7 integrin, CD50, CD54, and CD102 but not to CD62L.  Ref: 12640305 Transplantation, 2003
- Indeed, TGF beta induced Pyk2 phosphorylation was inhibited by monoclonal antibodies against VLA  [ **ITGA1** ]  4, VLA  [ **ITGA1** ]  5 and CD29  [ **ITGB1** ] .  Ref: 11683954 Immunology, 2001
- However, olopatadine had no effect on IL 5 induced CD49d CD29  [ **ITGB1** ]  VLA  [ **ITGA1** ]  4 expression.  Ref: 12046991 Jpn J Pharmacol, 2002
- Data obtained are presented in concurrence with Clusters of Thymic Epithelial Staining CTES classification for various monoclonal antibodies recognizing CD antigens CD1, CD1a, CD6, CD9, CD14, CD16, CD29  [ **ITGB1** ] , CD30, CD32, CD44, CD45RB, CD47, CD48, CD49a  [ **ITGA1** ] , CD49b, CD49c, CD49d, CD49e, CD49f, CD51, CD53, CD54, CD56, CD57, CD63, CD85, CD95, CD98, CD102, CD103, CD106, CD109, CD146, CD147, CD148, CD151, CD152, CD158a, CD158b, CD164, CD165, CD166 and for monoclonal antibodies 1B1 G7, A4, BD46, BLTZ, HP1C5, IND.64, M72, WU947 whose specifities are not yet defined.  Ref: 12725440 Ann Anat, 2003
- Surface integrin Mac 1 CD11b CD18 and VLA  [ **ITGA1** ]  4 CD49d CD29  [ **ITGB1** ]  expression on monocytes and the relative number of monocyte platelet complexes after in vitro activation were significantly elevated in patients with restenosis compared with patients without restenosis fluorescence intensities of 1425 76 vs 1195 7 7 7 vs 65 6 and 47 4 vs 29 3% for for Mac 1, VLA  [ **ITGA1** ]  4 and monocyte platelet complexes, respectively.  Ref: 11553019 Clin Exp Pharmacol Physiol, 2001
- We found that T h 1 cells derived from ovalbumin OVA specific TCR transgenic DO11.10 mice showed significantly higher levels of VLA  [ **ITGA1** ]  2 CD49b CD29  [ **ITGB1** ]  expression than T h 2 cells.  Ref: 12750354 Int Immunol, 2003
- Furthermore, eosinophil recruitment and activation appears to be mediated by a combination of VLA  [ **ITGA1** ]  4 CD49d CD29  [ **ITGB1** ]  and LFA 1 CD18 CD11a.  Ref: 12877819 Pulm Pharmacol Ther, 2003
- The data show that in the allergic rabbit model of asthma, VLA  [ **ITGA1** ]  4 CD49d CD29  [ **ITGB1** ]  only, is involved in the acute bronchoconstriction, suggesting an involvement of mast cell degranulation.  Ref: 12877819 Pulm Pharmacol Ther, 2003
- The up regulation of different adhesion molecules such as VLA  [ **ITGA1** ]  4 CD49d CD29  [ **ITGB1** ]  and ICAM 1 CD54 on the pulmonary endothelium and leukocytes, is a key event in sepsis induced lung injury leading to inflammatory tissue alterations.  Ref: 11355415 Int J Legal Med, 2001
- Flow cytometry was used to evaluate the expression rate of VLA  [ **ITGA1** ]  4 CD49d CD29  [ **ITGB1** ]  and VLA  [ **ITGA1** ]  5 CD49e CD29  [ **ITGB1** ]  and beta1 integrin activation ability in the transfected K562 cells.  Ref: 12844402 Zhongguo Shi Yan Xue Ye Xue Za Zhi, 2003
- Leukocyte function associated antigen LFA 1, alpha L beta 2, and CD11a CD18 and very late antigen VLA  [ **ITGA1** ]  4, alpha 4 beta 1, and CD49d CD29  [ **ITGB1** ]  are members of integrin type CAM that are predominantly involved in leukocyte trafficking and extravasation.  Ref: 11857637 Med Res Rev, 2002
- Post mortem markers of sepsis an immunohistochemical study using VLA  [ **ITGA1** ]  4 CD49d CD29  [ **ITGB1** ]  and ICAM 1 CD54 for the detection of sepsis induced lung injury.  Ref: 11355415 Int J Legal Med, 2001
